# Associations between vaping and self-reported respiratory symptoms in young people in Canada, England and the US

**DOI:** 10.1186/s12916-024-03428-6

**Published:** 2024-05-29

**Authors:** Leonie S. Brose, Jessica L. Reid, Debbie Robson, Ann McNeill, David Hammond

**Affiliations:** 1 Institute of Psychiatry, Psychology and Neuroscience (IoPPN), King’s College London, London, SE5 8BB UK; 2 School of Public Health Sciences, University of Waterloo, Waterloo, Canada

**Keywords:** Adolescents, Vaping, Surveys, Respiratory health

## Abstract

**Background:**

Prevalence of youth nicotine vaping has increased, heightening concerns around negative health effects. This study aimed to compare self-reported respiratory symptoms among youth by vaping behaviours.

**Methods:**

Participants (*n* = 39,214) aged 16–19 from the 2020 and 2021 International Tobacco Control Policy Evaluation Project (ITC) Youth Tobacco and Vaping Surveys (Canada, England, US). Weighted multivariable logistic regression assessed associations between reporting any of five respiratory symptoms in the past week (shortness of breath, wheezing, chest pain, phlegm, cough) and: past 30-day smoking and/or vaping; lifetime/current vaping. Among past-30-day vapers (*n* = 4644), we assessed associations between symptoms and vaping frequency, use of nicotine salts, usual flavour and device type(s).

**Results:**

Overall, 27.8% reported experiencing any of the five respiratory symptoms. Compared with youth who had only vaped, those who had only smoked had similar odds of symptoms [adjusted odds ratio, OR (95% confidence interval, CI): 0.97 (0.85–1.10)], those who both smoked and vaped had higher odds [1.26 (1.12–1.42)], and those who had done neither, lower odds [0.67 (0.61–0.72)]. Compared with those who had never vaped, past use, experimentation and current regular or occasional use were all associated with higher odds. Reporting usually using nicotine salts was associated with higher odds of symptoms [1.43 (1.22–1.68)] than non-salt but was often uncertain. Compared with tobacco flavour (including with menthol), menthol/mint and sweets flavours were associated with similar odds; fruit [1.44 (1.07–1.93)], multiple [1.76 (1.30–2.39)] and ‘other’ [2.14 (1.45–3.16)] flavours with higher odds. All device types were associated with similar odds.

**Conclusions:**

Among youth, vaping was associated with increased reporting of past-week respiratory symptoms. Among those who vaped, some flavour types and potentially nicotine salts were associated with respiratory symptoms.

**Supplementary Information:**

The online version contains supplementary material available at 10.1186/s12916-024-03428-6.

## Background

Over the last decade, vaping prevalence has increased substantially among adolescents in the United States (US), Canada and England, while cigarette smoking has decreased or changed little [1–5]. Among the many adverse health effects of smoking are respiratory symptoms and diseases; young people who smoke have increased risks of cough and phlegm production, respiratory illnesses, potential retardation in the rate of lung growth and the level of maximum lung function [6]. For people who smoke, switching to vaping reduces health risk [3, 7], and while there is strong evidence that nicotine vaping products are effective for assisting adults with smoking cessation [8], regular use by young people, particularly those who would not have smoked, raises concerns around health harms independent of smoking. People who vape are exposed to fine and ultrafine particles and potentially harmful constituents in vape aerosol which could be associated with long-term conditions; however, there is a lack of evidence, particularly among those without a history of smoking [3, 7]. A systematic review of objective markers of the effect of vaping on respiratory health found insufficient evidence on whether vaping has any impact on lung function after acute, short- to medium-, or long-term exposure [3]. Studies using the US PATH survey [9] which asks about wheezing and cough in the past year found somewhat conflicting results about associations between vaping and self-reported past-year respiratory symptoms among youth aged 12–17 and young adults [10–14], partly explained by different approaches to accounting for cigarette smoking. In another cross-sectional survey of young adults, cannabis vaping was associated with respiratory symptoms while there were no significant associations for nicotine vaping [15], highlighting the importance of considering a range of other inhaled products. However, another longitudinal survey in young adults found vaping was associated with symptoms independent of combustible cannabis and tobacco exposure [16]. There is little evidence from other countries, and the existing evidence is limited because surveys asked about symptoms in the past year when, particularly among youth, vaping and smoking behaviour may change rapidly. Additionally, previous smoking was generally not considered, and vaping measures grouped all past 30-day vaping (regardless of vaping once or every day) or even past-year vaping whereas a dose–response relationship can be expected.

Different types of flavours and devices may affect respiratory symptoms either directly or via associated differences in behaviour such as increased depth of inhalation and increased frequency of use. Similarly, vaping liquids may use salt or free-base nicotine formulations, and it has been hypothesised that salts may enable deeper inhalation and increased use [17]. Evidence is scare however; one survey of young people in the US who had vaped in the past 30 days found that associations with respiratory symptoms did not differ by the type of device used most frequently (pod, pen/tank, disposable, mod) [18]; there is scarce evidence on other characteristics and from countries outside the US. Restrictions on flavours or devices are being considered or introduced with the aim to reduce youth vaping [19, 20].

### Research aim

The research aim is to assess self-reported respiratory symptoms in young people in Canada, England and the US by smoking and vaping behaviour and country.

#### Hypotheses

1. Current vaping will be associated with lower odds of respiratory symptoms than current smoking and higher odds than not smoking or vaping.
2. More frequent vaping (lifetime/current) will be associated with higher odds of respiratory symptoms than less frequent vaping, independent of smoking status.
3. Vaping nicotine salts will be associated with higher odds of respiratory symptoms compared with vaping other forms of nicotine; this will at least partly be explained by frequency of vaping.
4. Different types of flavours or types of devices will not be associated with respiratory symptoms independent of frequency, nicotine type and smoking status.
5. There will be no country differences and no interactions between country and vaping, or country and vaping characteristics on respiratory symptoms.

## Methods

Additional detail is available in the pre-registration at https://osf.io/9d3a8.

### Design

The ITC Youth Tobacco and Vaping Survey is an online survey examining use of tobacco and vaping products among youth aged 16–19 in three countries with different policy environments: Canada, England and the US. The sample was recruited from the Nielsen Consumer Insights Global Panel and their partner panels. Invitations were emailed to panellists, including those with children aged 16–19. After eligibility screening, potential respondents (and parents, where applicable) were provided with information about the study and asked for consent. Remuneration was in accordance with the panels’ usual incentive structure. The present analysis included pooled data from three cross-sectional survey waves conducted in 2020 and 2021 with new respondents at each wave. Separately for each country, post-stratification sample weights were constructed based on population estimates for sociodemographic variables, variables (age, sex, geographic region, and race/ethnicity (US only), and calibrated to wave 1 proportions for student status and academic grades, as well as the trend over time for past-30-day smoking (for the US and Canada), enhancing applicability of results to the general population of 16–19-year-olds. Technical reports provide further details [21–23].

### Sample

The survey waves included *n* = 42,533 participants. We excluded participants with asthma (*n* = 2798, 6.6%) or missing data on vaping or smoking (*n* = 521, 1.2%), leaving an unweighted *n* = 39,214 for analysis. Analyses of those who had vaped in the past 30 days included unweighted *n* = 4644.

### Measures

All measures except country and survey wave were self-reported. Socio-demographics included *Country* (Canada, England, US), *Age group* (16–17; 18–19 years), *Race/ethnicity* (country-specific questions/responses collapsed into White only; Everyone else), *Sex* (assigned at birth or inferred from gender: Male, Female); and *Family socio-economic status* (Not meeting basic expenses; Just meeting basic expenses; Meeting needs with a little left over; Living comfortably; Don’t know; Refused, with final two options combined for analysis). *Survey wave* (August 2020, February/March 2021, August/September 2021) was also included.

#### Outcomes

The *primary outcome* was reporting *any* of (i) shortness of breath, (ii) wheezing, (iii) chest pain, (iv) phlegm, or (v) cough, in the past week [24, 25]. Don’t know and refused responses were categorised as no (1.8%, shortness of breath to 3.1%, phlegm). *Secondary outcomes* were each of the five symptoms individually.

#### Smoking and vaping

*Past-30-day smoking and/or vaping*: Four categories were derived from questions about ever use and, among those who had ever used, most recent use: Vaping only, Smoking only, Smoking and vaping, Neither smoking nor vaping.

*Lifetime/current smoking* was based on several questions (ever use, lifetime use, most recent use) and coded into five exclusive categories: ≥ 20 days in past 30 days (smoked at least 100 cigarettes in their lifetime and on at least 20 of the past 30 days), < 20 days in past 30 days (smoked at least 100 cigarettes in their lifetime and on fewer than 20 of the past 30 days), Former smoking (at least 100 cigarettes in lifetime, did not smoke in the past 30 days), Experimental smoking (fewer than 100 cigarettes in lifetime, regardless of recency), Never smoked (never tried cigarettes).

*Lifetime/current vaping* was derived from several questions (ever use, lifetime use, most recent use) and coded into five exclusive categories: ≥ 20 days in past 30 days (vaped on at least 20 of the past 30 days), < 20 days in past 30 days (vaped on more than 10 days in their lifetime and on fewer than 20 of the past 30 days), Former vaping (vaped on more than 10 days in their lifetime, not in the past 30 days), Experimental vaping (vaped on up to 10 days in their lifetime, regardless of recency), Never vaped (never tried vaping).

Among those who had vaped in the past 30 days, additional characteristics were assessed. *Number of days vaped*: continuous variable for number of days in the past 30 (0–30). *Nicotine type*: Respondents reporting awareness of nicotine salts were asked if the e-cigarettes, cartridges, pods, or e-liquids they currently use contain nicotine salts, and responses were recoded into yes versus other responses, including don’t know (11.0% of those aware), refused (0%) and unaware of nicotine salts (50.0% of those who had vaped in the past 30 days). Because a large proportion were unaware, an additional version of the variable included only those aware of nicotine salts. *Flavour type*: ‘In the LAST 30 DAYS, which of these flavours did you use MOST OFTEN?’, with original 12 response options grouped into: Tobacco (including Mix of tobacco and menthol); Menthol or mint; Fruit; Sweets; Other/unknown (including 1.2% Don’t know and 0.2% Refused); Multiple. *Device type*: ‘Which of the following types of e-cigarettes/vaping devices do you currently use most often?’ Disposable; Pre-filled cartridges/pods; Tank; Multiple, unknown (2.3% did not know/refused).

Using questions about heated tobacco products, cannabis vaping or smoking and other combustible tobacco product use, *other (inhaled) product use* in the last 30 days was coded as follows: None; One other type; Two or three types.

### Analysis

All analyses used weighted data and were conducted in IBM SPSS 27 and 29; 95% confidence intervals excluding 1.00 were interpreted as significant. Models were adjusted as per the pre-registration.

#### Sample description

Socio-demographics, smoking and vaping, vaping characteristics and other product use were described using proportions. Proportions reporting any symptoms were reported overall, by characteristics for the full sample and for those who had not used other products.

#### Analyses addressing hypotheses

Bivariate logistic regression assessed associations between the primary outcome and combined past-30-day smoking and/or vaping; multivariable logistic regression also included socio-demographics, wave and other product use.
(I) Bivariate logistic regression assessed the association between lifetime/current vaping and the primary outcome, followed by multivariable logistic regression adjusting for lifetime/current smoking, socio-demographics, wave and other product use. (II) Among respondents who had vaped in the past 30 days, bivariate logistic regression assessed the association between number of days vaped and the primary outcome, and multivariable logistic regressions adjusted for lifetime/current smoking, socio-demographics, wave and other product use.
Among respondents who had vaped in the past 30 days, bivariate logistic regression assessed associations between primary outcome and nicotine type; multivariable logistic regression included number of days vaped, lifetime/current smoking, socio-demographics, wave and other product use. Because a large proportion of respondents were not aware of nicotine salts or unsure about their use, analyses were repeated including only those who reported awareness.
Among respondents who had vaped in the past 30 days, separate bivariate logistic regression models assessed associations between primary outcome and flavours or type of device; separate multivariable logistic regressions included number of days vaped, lifetime/current smoking, socio-demographics, wave, other product use and nicotine type. As reference categories, we used the most commonly used device type and tobacco flavour, which is usually allowed where flavours are restricted.
Exploratory (pre-specified) analyses assessed the associations between country and the primary outcome in bivariate logistic regressions with England as reference category, followed by models adjusting for socio-demographics and wave and models including an interaction for country and the main variable of interest for each hypothesis: country * (i) past 30-day smoking and/or vaping, (ii) lifetime/current vaping, (iii) nicotine type, (iv) flavour type, (v) device type.

##### Sensitivity and supplementary analyses

I. For all hypotheses, sensitivity analyses repeated the adjusted analyses for the primary outcome but excluding respondents using other products.
II. Data were collected during the Coronavirus disease 2019 (COVID-19) pandemic; in the 2021 waves, respondents were asked if they thought they had had COVID-19 in the last 2 weeks. As COVID-19 generally causes respiratory symptoms, we ran additional sensitivity analyses restricted to the 2021 waves and adjusting for COVID for all hypotheses. Information on any other respiratory infections was not available.
III. Supplementary analyses assessed associations for each of the secondary outcomes using the same models as for the primary analyses.

#### Changes from pre-registration

Participants with asthma were excluded similar to other publications [14]. For ethnicity, 1.4% responding ‘don’t know’ or refused to answer were included in ‘Everyone else’. For nicotine type, two versions of the variable were used due to the large proportion not aware of nicotine salts. For hypothesis 5, vaping characteristics differed between countries, so that adjusting for those characteristics may have biased results and we ran models as described. Analysis including COVID-19 information were added.

## Results

### Sample

Weighted sample characteristics are shown in Table 1 (unweighted in Additional file 1: Table S1). By design, it comprised similar proportions from all three waves, age groups, sex and country. About two thirds considered themselves white, and about one quarter reported their family not or just being able to meet basic expenses. In the past 30 days, most had not smoked or vaped, few had smoked or vaped on at least 20 days, and 16.3% had used one or more other inhaled product, mostly cannabis (12.2%; 6.6% other tobacco; 0.9% heated tobacco).

**Table 1:** Sample description and respiratory symptoms by characteristic for the full sample and for those who had not used other inhaled products in the past 30 days (weighted data)

Characteristic	Sample description		Reported any respiratory symptoms			
			**Within full sample**^a^		**Within those not using other products**^**c**^	
	**%**	***n***	**%**	***n***	**%**	***n***
**Age group (years)**						
16–17	49.3	19,452	24.6	4776	22.0	3764
18–19	50.7	19,982	31.1	6205	26.9	4285
**Sex**						
Male	51.6	20,347	25.8	5245	21.6	3649
Female	48.4	19,087	30.0	5735	27.2	4400
**Country**						
Canada	32.1	12,644	25.1	3177	21.3	2215
England	30.0	11,815	28.3	3340	24.8	2531
US	38.0	14,976	29.8	4463	26.6	3303
**Race/ethnicity**						
White	65.4	25,786	26.6	6855	23.1	4963
Everyone else	34.6	13,648	30.2	4125	26.8	3086
**Perceived family socio-economic situation**						
Not meeting basic expenses	2.9	1141	38.6	441	31.5	235
Just meeting basic expenses	20.4	8045	33.1	2665	28.5	1788
Meeting needs with a little left over	34.5	13,587	27.0	3674	23.8	2759
Living comfortably	37.1	14,639	25.6	3753	23.0	2893
Don’t know/refused	5.1	2023	22.1	447	20.2	373
**Past 30-day smoking and/or vaping**						
Vaped only	8.7	3428	40.1	1375	33.4	597
Smoked only	4.0	1564	38.8	607	30.5	249
Smoked and vaped	4.5	1783	48.8	870	33.5	207
Neither	82.8	32,659	24.9	8129	23.5	6995
**Lifetime/current smoking**						
≥ 20 days in past 30 days	2.3	910	52.4	477	38.2	125
< 20 days in past 30 days	1.4	555	46.3	257	33.7	70
Smoked in the past	1.0	403	40.2	162	34.4	73
Experimented in the past	25.2	9923	33.0	3278	26.9	1815
Never smoked	70.1	27,642	24.6	6807	23.4	5966
**Lifetime/current vaping**						
≥ 20 days in past 30 days	4.4	1745	47.9	836	40.5	284
< 20 days in past 30 days	5.9	2317	42.8	992	31.6	332
Vaped in the past	5.8	2290	35.2	805	28.4	398
Experimented in the past	20.4	8052	31.7	2552	29.3	1846
Never vaped	63.5	25,032	23.2	5796	22.0	5188
**Other inhaled product use in past 30 days**^**b**^						
None	83.8	33,029	24.4	8049	n/a	n/a
1 type of product	13.1	5159	42.7	2202	n/a	n/a
2 or 3 types of products	3.2	1247	58.5	730	n/a	n/a
**Survey wave**						
August 2020	34.3	13,529	28.5	3860	25.2	2862
February/March 2021	33.1	13,045	26.5	3454	23.0	2497
August/September 2021	32.6	12,861	28.5	3666	24.9	2690

Columns 2 and 3 (Sample description) show the proportion and number reporting each characteristic such as being aged 16 or 17, and the subgroups will add up to 100% (allowing for rounding). Columns 4 to 7 (Reported any respiratory symptoms) show the proportion and number within each subgroup who reported symptoms, e.g. the proportion of those aged 16 or 17 who reported experiencing any respiratory symptoms in the past week; therefore, the subgroups will not add up to 100% in these columns

^a^Unweighted *n* = 39,214

^b^Proportion reporting past-30-day use in the full sample

^c^Unweighted *n* = 32,274. This is the sample of people who reported no use of heated tobacco, smoking or vaping cannabis or other combustible tobacco products

Overall, 27.8% (*n* = 10,980) reported any respiratory symptoms in the past week, most commonly cough (16.1%), followed by shortness of breath (10.0%), chest pain (10.0%), phlegm (8.5%) and wheezing (2.6%). Among those who had not used any other products in the past month, 24.4% (*n* = 8049) reported any symptoms. There was some variation by socio-demographics, smoking, vaping and other product use (Table 1, Additional file 1: Table S2). In the 2021 waves, 3.3% thought they had had COVID-19 in the past 2 weeks, 2.8% did not know and 0.3% refused to say. Among those reporting COVID-19, 72.3% reported respiratory symptoms.

Among those who had vaped in the past 30 days (unweighted *n* = 4644), the median number of days vaped was 12 (interquartile range 3–30). Among those who had vaped and were aware of nicotine salts (unweighted *n* = 1874), just over half (53.5%) reported using salts; among all those who had vaped in the past 30 days, this was 21.6%. Cartridge/pod device types and fruit flavours were the most used, multiple devices and tobacco flavours the least (Table 2, unweighted in Additional file 1: Table S3).

**Table 2:** Vaping product characteristics and respiratory symptoms by characteristic among those who had vaped in the past 30 days (weighted data)

Vaping product characteristic	Sample description		Reported any respiratory symptoms			
			**Within full sample**		**Within those not using other products**	
	**%**	***n***	**%**	***n***	**%**	***n***
**Nicotine type**						
Non-salt, unknown	78.4	3425	40.4	1382	31.2	534
Salt	21.6	944	53.8	508	49.7	143
**Flavour type**						
Fruit	48.7	2125	41.3	878	33.7	367
Multiple	24.3	1051	51.3	539	43.2	147
Menthol or mint	13.9	605	38.0	230	26.5	76
Tobacco (incl. menthol tobacco)	5.7	249	34.3	85	26.1	31
Other, unknown	4.9	215	49.5	106	33.6	36
Sweets	2.8	123	40.7	50	32.3	20
**Device type**						
Pre-filled cartridge/pod	36.6	1599	41.3	660	32.3	245
Tank	30.2	1320	41.4	546	34.0	239
Disposable	17.5	763	46.0	351	33.2	102
Multiple, unknown	15.7	686	48.5	333	38.7	91

Columns 2 and 3 (Sample description) show the proportion and number reporting each characteristic such as using nicotine salts, and the subgroups will add up to 100% (allowing for rounding). Columns 4 to 7 (Reported any respiratory symptoms) show the proportion and number within each subgroup who reported symptoms, e.g. the proportion of those using nicotine salts who reported experiencing any respiratory symptoms in the past week; therefore, the subgroups will not add up to 100% in these columns

Unweighted *n* = 4644 for full sample, *n* = 2093 for those not using other products (those who reported no use of heated tobacco, smoking or vaping cannabis or other combustible tobacco products)

### Hypothesis 1. Past-30-day smoking and/or vaping

Compared with those who had only vaped, those who had both smoked and vaped had higher odds of experiencing any symptoms, whereas those who had neither smoked nor vaped had lower odds of symptoms and those who had only smoked were similar to those who had only vaped. These associations were found in unadjusted and adjusted analyses (Table 3).

**Table 3:** Associations between (a) past-30-day smoking and/or vaping and (b) lifetime/current vaping and any respiratory symptoms (separate models) (weighted data)

	**Unadjusted OR (95% CI)**	***P*-value**	**Adjusted OR (95% CI)**	***P*-value**
**a) Past 30-day smoking and/or vaping**^**a**^				
Vaped only	Ref		Ref	
Smoked only	0.95 (0.84–1.07)	0.365	0.97 (0.85–1.10)	0.607
Smoked and vaped	1.42 (1.27–1.60)	< 0.001	1.26 (1.12–1.42)	< 0.001
Neither	0.50 (0.46–0.53)	< 0.001	0.67 (0.61–0.72)	< 0.001
**b) Lifetime/current vaping**^**b**^				
≥ 20 days in past 30 days	3.06 (2.77–3.37)	< 0.001	1.98 (1.77–2.23)	< 0.001
< 20 days in past 30 days	2.48 (2.28–2.71)	< 0.001	1.62 (1.46–1.79)	< 0.001
Vaped in the past	1.80 (1.65–1.97)	< 0.001	1.32 (1.19–1.46)	< 0.001
Experimented in the past	1.54 (1.46–1.63)	< 0.001	1.30 (1.22–1.38)	< 0.001
Never vaped	Ref		Ref	

Unweighted *n* = 39,214. Ref: Reference category

^a^Adjusted analysis included age group, sex, country, ethnicity, perceived family socio-economic status, use of other inhaled products, wave

^b﻿^Adjusted analysis included age group, sex, country, ethnicity, perceived family socio-economic status, use of other inhaled products, wave, lifetime/current smoking

Sensitivity analyses including only those who had not used other inhaled products found no difference between those who had both smoked and vaped compared with those who had only vaped; the other associations remained similar (Additional file 1: Table S4). Results from sensitivity analyses in the latter two waves and adjusting for COVID-19 remained very similar to the primary analysis (Additional file 1: Table S4), and associations with individual symptoms generally also agreed (Additional file 1: Table S5).

### Hypothesis 2. Lifetime/current vaping

Compared with those who had never vaped, all other groups were more likely to have experienced respiratory symptoms, with those who had vaped on at least 20 of the past 30 days most likely to report symptoms, in unadjusted and adjusted analysis (Table 3). Results from sensitivity analyses including only those who had not used other inhaled products, or adjusting for COVID-19, were also similar (Additional file 1: Table S4), as were most associations with individual symptoms, except that wheezing differed less between groups (Additional file 1: Table S5).

Among those who had vaped in the past 30 days, each additional day vaping was associated with a small but statistically significant increase in the odds of experiencing respiratory symptoms (unadjusted OR (95% CI): 1.02 (1.01–1.02), *p* < 0.001; adjusted OR (95% CI): 1.01 (1.01–1.02), *p* < 0.001. This association was also found in the sensitivity analyses (Additional file 1: Table S4) and for individual symptoms (Additional file 1: Table S5).

### Hypothesis 3. Nicotine type

Among respondents who had vaped in the past 30 days, usual nicotine salt use was associated with higher odds of respiratory symptoms, and this association remained when adjusting for other variables (Table 4) and in the sensitivity analyses excluding those using other products or adjusting for COVID-19 (Additional file 1: Table S6). When including only those reporting awareness of nicotine salts, an association with symptoms was found in the unadjusted analyses [OR (95% CI): 1.21 (1.00–1.46), *p* = 0.047], but not in adjusted analysis [OR (95% CI): 1.14 (0.93–1.38), *p* = 0.205, unweighted *n* = 1874]. Those categorised as using nicotine salts had higher odds of reporting each of the five symptoms (Additional file 1: Table S7).

**Table 4:** Associations between vaping product characteristics and any respiratory symptoms (separate models) among those who had vaped in past 30 days (weighted data)

Vaping product characteristic	Unadjusted OR (95% CI)	*P*-value	Adjusted OR (95% CI)	*P*-value
**Nicotine type**^**a**^				
Non-salt, unknown	Ref		Ref	
Salt	1.72 (1.49–1.99)	< 0.001	1.43 (1.22–1.68)	< 0.001
**Flavour type**^**b**^				
Tobacco (incl. menthol tobacco)	Ref		Ref	
Menthol or mint	1.18 (0.87–1.61)	0.294	1.26 (0.91–1.74)	0.167
Fruit	1.35 (1.03–1.78)	0.032	1.44 (1.07–1.93)	0.014
Sweets	1.31 (0.84–2.05)	0.228	1.44 (0.91–2.29)	0.119
Other, unknown	1.88 (1.29–2.73)	< 0.001	2.14 (1.45–3.16)	< 0.001
Multiple	2.02 (1.52–2.70)	< 0.001	1.76 (1.30–2.39)	< 0.001
**Device type**^**c**^				
Pre-filled cartridge/pod	Ref		Ref	
Disposable	1.21 (1.02–1.44)	0.031	1.15 (0.95–1.37)	0.145
Tank	1.00 (0.86–1.16)	0.981	0.97 (0.82–1.13)	0.669
Multiple, unknown	1.34 (1.12–1.60)	0.001	1.09 (0.90–1.31)	0.396

Unweighted *n* = 4644

^a^Adjusted analysis included age group, sex, country, ethnicity, perceived family socio-economic status, use of other inhaled products, wave, lifetime/current smoking, number of days vaped

^b^Adjusted analysis included age group, sex, country, ethnicity, perceived family socio-economic status, use of other inhaled products, wave, lifetime/current smoking, number of days vaped, nicotine type

^c^Adjusted analysis included age group, sex, country, ethnicity, perceived family socio-economic status, use of other inhaled products, wave, lifetime/current smoking, number of days vaped, nicotine type

### Hypothesis 4. Flavours and devices

Compared with tobacco flavours, menthol/mint and sweet flavours were associated with similar odds of symptoms; use of fruit flavours, ‘other/unknown’ and multiple flavours were all associated with higher odds of symptoms, and adjustment for other variables had little effect (Table 4). In the sensitivity analysis excluding those who had used other products, only use of multiple flavour types was associated with higher odds of symptoms (Additional file 1: Table S6). In the sensitivity analysis including COVID-19, in addition to fruit, other/unknown and multiple flavours, sweets flavours were also associated with higher odds of reporting symptoms than tobacco flavours (Additional file 1: Table S6). In analysis of individual symptoms, compared with tobacco flavours, all flavour types were associated with higher odds of cough, and ‘other/unknown’ flavour types were also associated with higher odds for chest pain; there were no significant associations with shortness of breath, wheezing or phlegm (Additional file 1: Table S7).

Compared with pre-filled cartridge/pod models, use of disposable, multiple or unknown device types was associated with higher odds of symptoms only in unadjusted analysis, and the odds for tank device types were similar to those for pre-filled cartridge/pod devices (Table 4). The sensitivity analyses found no significant associations (Additional file 1: Table S6). Compared with pre-filled cartridge/pod devices, disposable devices were associated with higher odds of shortness of breath, chest pain and phlegm; using multiple/unknown devices was also associated with higher odds of chest pain; for tanks, odds of each symptom were not different from pre-filled cartridge/pods. For wheezing and cough, there were no significant associations with device type (Additional file 1: Table S7).

### Hypothesis 5. Country

The odds of symptoms were lower for Canada and higher for the US than for England among all respondents; among youth who had vaped in the past 30 days, the three countries were similar (Table 5). Sensitivity analyses were similar (Additional file 1: Table S8).

**Table 5:** Associations between country and any respiratory symptoms (weighted data)

	**Unadjusted OR (95% CI)**	***p*-value**	**Adjusted OR (95% CI)**	***P*-value**
**Country, full sample**^**a**^				
England	Ref		Ref	
Canada	0.85 (0.81–0.90)	< 0.001	0.82 (0.78–0.95)	< 0.001
US	1.08 (1.02–1.14)	0.006	1.07 (1.01–1.13)	0.014
**Country, vaped in past 30 days**^**b**^				
England	Ref		Ref	
Canada	1.03 (0.89–1.20)	0.684	1.01 (0.87–1.18)	0.855
US	0.93 (0.80–1.08)	0.343	0.92 (0.79–1.07)	0.262

Adjusted analysis included age group, sex, country, ethnicity, perceived family socio-economic status, wave

^a﻿^Unweighted *n* = 39,214

^b^Unweighted *n* = 4644

We found an interaction for country and past-30-day smoking and/or vaping (overall *p* < 0.001, Additional file 1: Table S9), with odds of symptoms for those in Canada who had neither vaped or smoked lower than for those who had vaped compared with England. This is in line with the lower rates of symptoms reported in Canada among all respondents (the vast majority of whom had neither vaped nor smoked).

There was also a significant interaction for lifetime/current vaping (*p* < 0.001, Additional file 1: Table S9). In Canada, there was a larger difference between those who had vaped on 20 or more of the past 30 days and those who had never vaped than in England. In the US, the difference between those who had never vaped compared with those who had vaped on less than 20 of the past 30 days, had vaped in the past or had experimented in the past was smaller than in England. This is in line with the overall higher rates of symptoms reported among all (mostly never vaping) respondents in the US.

There was an overall significant interaction for nicotine type and country (*p* = 0.003), but none of the individual contrasts indicated a difference. The overall interactions for flavours (*p* = 0.257) or devices (*p* = 0.226) were not significant.

For individual symptoms, compared with England, Canada was associated with lower odds for all five symptoms, the US with lower odds for wheezing and higher odds for chest pain and cough. In those who had vaped in the past 30 days, the only association was for wheezing, with lower odds in the US (Additional file 1: Table S10).

## Discussion

In this survey study of young people in Canada, England and the US, our hypothesis that current vaping is associated with lower odds of respiratory symptoms than current smoking and higher odds than not smoking or vaping was partially supported with lack of evidence for a difference between vaping and smoking. The hypothesis that frequency of vaping would be associated with symptoms was supported both when using past-30-day frequency and lifetime/current vaping exposure. Among youth who vaped in the past 30 days, some characteristics of vaping products were also associated with respiratory symptoms, partially supporting our hypotheses. Using nicotine salts may be associated with increased symptoms, although this result was sensitive to how we categorised those who vaped but had not previously heard of nicotine salts. It is worth highlighting that many respondents were unsure about the type of nicotine in their usual vaping products, so any self-reported data on nicotine type in this and other studies should be interpreted with caution. While there was no evidence of differential associations by usual device type when using any symptoms as outcome, disposables were associated with three of five individual symptoms. Respondents who reported usually using multiple flavours were consistently more likely to report symptoms, while fruit flavours and ‘other’ were only associated if not excluding use of other products. Findings suggested no clear differences in respiratory symptoms across the three countries among young people who vape.

By including youth from Canada and England as well as the US, we were able to extend existing evidence and show that associations for vaping were generally similar across countries. We were also able to show for the first time in youth surveys a clear relationship between vaping frequency and respiratory symptoms. The present study provides new evidence on whether vaping product characteristics, including device types, flavours or nicotine types, show differences in associations with respiratory symptoms, extending previous studies which assessed general associations between vaping and symptoms. Previous studies, primarily using the US PATH survey, mostly found no associations between vaping and acute respiratory symptoms in young people once taking into account smoking status [9–13], whereas in this survey, associations persisted when adjusting for smoking and when excluding those who smoked or vaped other products. The present survey data were collected more recently (2020–2021 compared with 2015–2–18), and both the vaping product market and patterns in vaping behaviour have changed during this time [3, 26]. In addition, the present survey asked about past-week, compared with past-year, symptoms. However, the present findings are in line with a longitudinal study which used 2015–2018 data and assessed associations between past-year symptoms and past-30-day vaping [16].

The study had some limitations. The surveys were cross-sectional, measuring vaping/smoking behaviour at the same time as respiratory symptoms, so associations do not mean that there are causal links, and it is not clear if the vaping/smoking occurred before the onset of any respiratory symptoms. However, the use of past-week rather than past-year symptoms increases confidence in this finding. The surveys did not assess all factors that influence respiratory symptoms, some of which may be more common in people who are vaping or smoking, thus potentially inflating the found associations. While we were able to adjust for self-reported COVID-19, other infections may also have caused symptoms, although for this to have affected the results would require differential infection rates by vaping or smoking status. It is possible for example that infection risk is increased through sharing of vapes or cigarettes. Other omissions include second-hand exposure to smoking or vaping and air quality in general. However, while not a comprehensive assessment, a measure of familial poverty was included which is associated with exposure to poorer air quality [27].

The outcome measure had been used previously to assess symptoms but has limitations. It relied on recall and self-report. Self-reported measures are no replacement for clinically verified assessment but serve as informative indicators in population surveys, particularly for comparing trends over time or between subgroups. The measure used may have been interpreted by respondents to include even a single cough or shortness of breath in situations where this would not indicate respiratory problems; the heightened focus on respiratory symptoms during the COVID-19 pandemic could also have increased respondents' attention to symptoms. These points would explain the overall high prevalence of symptoms, including in those not vaping or smoking, which indicates that these are not all serious symptoms or leading to clinically significant health problems. For some participants, reported symptoms may also have been acute responses to new exposure to vaping [28]. It could also be speculated if coverage of a 2019 outbreak of lung injuries in the US [29] had heightened the focus on respiratory symptoms among people who vape which would have inflated reporting among this group. However, there is no evidence that recall of symptoms or interpretation of the measure would differ between young people who vape or smoke.

Generally, characteristics that enable more frequent and prolonged use of vaping products are likely to exacerbate any negative respiratory effects, so devices and flavours may be proxy measures for greater exposure; however, by including frequency of use in analyses, we mitigated against this impacting results. We were not able to assess constituents of devices beyond the general categorisation into device types; for example, we do not know the proportion using more recent disposable types, which grew rapidly between 2021 and 2022 and often contain nicotine salts [26]. Similarly, while we assessed nicotine type, confidence in the results is limited by large proportions of youth who vaped not being aware of the type of nicotine used and associations not being clear when including only those who knew about nicotine salt. Absence of other information on constituents and composition of liquids further limits the findings. While assessing flavour types provides novel evidence, there is a wide range of specific flavours from different manufacturers available and different flavour types contain overlapping flavourings, both potentially masking effects of specific flavours. We were not able to assess any associations with nicotine strength in vaping liquids or amount of nicotine absorbed by the user, which may both affect vaping frequency and intensity and thereby symptom development. In England and more recently Canada, maximum nicotine strength is lower (2%) than what is commonly used in the US, so nicotine strength groupings would largely have aligned with country. The prevalence of recent cannabis use (12%) likely still underestimates actual use, particularly as it remains illegal in England and parts of the US, which may have led to overestimating the impact of vaping as some symptoms may be due to cannabis use. A clear strength of this analysis is the use of large national samples (enhanced by weighting) across three countries with different regulations and prevalence of youth vaping and smoking. The survey also included an assessment of smoking history and use of other inhaled products to assess the influence of wider product use rather than vaping in isolation.

Future research should assess respiratory health using standardised clinical tests, biomarkers of potential harm and verified long-term health outcomes. Inclusion of other exposures that can increase respiratory problems among youth is also needed. Improved recording of constituents of vaping liquids and assessment of effects of individual and combined constituents on respiratory health would help identify and remove potentially harmful constituents.

## Conclusions

Among youth, vaping was associated with increased reporting of past-week respiratory symptoms. Among those who vaped, some flavour types and potentially nicotine salts were associated with respiratory symptoms.

### Supplementary Information

Additional file 1: Table S1. Sample description and respiratory symptoms by characteristic for the full sample and for those who had not used other inhaled products in the past 30 days (unweighted data). Table S2. Respiratory symptoms in the past week broken down by other product use, weighted *n* (%). Table S3. Vaping product characteristics and respiratory symptoms by characteristic among those who had vaped in the past 30 days (unweighted data). Table S4. Associations between past-30-day smoking and/or vaping, lifetime/current vaping, number of days vaped in the past 30 days and any respiratory symptoms (weighted data). Table S5. Associations between past-30-day smoking and vaping, lifetime/current vaping, number of days vaped in the past 30 days and individual respiratory symptoms (weighted data). Table S6. Associations between vaping characteristics and any respiratory symptoms (weighted data). Table S7. Associations between vaping characteristics and individual respiratory symptoms (weighted data). Table S8. Sensitivity analysis. Associations between country and any respiratory symptoms for the full sample and those who had vaped in the past 30 days (weighted data). Table S9. Interaction models for country (weighted data). Table S10. Supplementary analysis. Associations between country and individual respiratory symptoms for the full sample and those who had vaped in the past 30 days (weighted data).

## Data Availability

Deidentified study data may be made available on request to researchers who submit a proposal that is approved by the principal investigator. Proposals should be submitted to David Hammond (dhammond@uwaterloo.ca).

## Abbreviations

CI: Confidence interval
COVID-19: Coronavirus disease 2019
ITC: International Tobacco Control Policy Evaluation Project
NIHR: National Institute for Health and Care Research
OR: Odds ratio
PATH: Population Assessment of Tobacco and Health
Ref: Reference category
US: United States
