# Supplementary material for: Associations between vaping and self-reported respiratory symptoms in young people in Canada, England and the US
Source: BMC Med. 2024 May 29;22:213. doi: 10.1186/s12916-024-03428-6 (PMC11134717; doi:10.1186/s12916-024-03428-6)
Supplement: Supplementary file 1 — Additional file 1: Table S1. Sample description and respiratory symptoms by characteristic for the full sample and for those who had not used other inhaled products in the past 30 days (unweighted data). Table S2. Respiratory symptoms in the past week broken down by other product use, weighted n (%). Table S3. Vaping product characteristics and respiratory symptoms by characteristic among those who had vaped in the past 30 days (unweighted data). Table S4. Associations between past-30-day smoking and/or vaping, lifetime/current vaping, number of days vaped in the past 30 days and any respiratory symptoms (weighted data). Table S5. Associations between past-30-day smoking and vaping, lifetime/current vaping, number of days vaped in the past 30 days and individual respiratory symptoms (weighted data). Table S6. Associations between vaping characteristics and any respiratory symptoms (weighted data). Table S7. Associations between vaping characteristics and individual respiratory symptoms (weighted data). Table S8. Sensitivity analysis. Associations between country and any respiratory symptoms for the full sample and those who had vaped in the past 30 days (weighted data). Table S9. Interaction models for country (weighted data). Table S10. Supplementary analysis. Associations between country and individual respiratory symptoms for the full sample and those who had vaped in the past 30 days (weighted data). [file 12916_2024_3428_MOESM1_ESM.docx]

Associations between vaping and respiratory symptoms in young people in Canada, England and the US – Supplementary material

# Leonie S Brose, Jessica L Reid, Debbie Robson, Ann McNeill, David Hammond

**Table S1. Sample description and respiratory symptoms by characteristic for the full sample and for those who had not used other inhaled products in the past 30 days (unweighted data).**

| **Characteristic** | **Sample description, N=39,214** | | **Reported any respiratory symptoms** | | | |
| --- | --- | --- | --- | --- | --- | --- |
|  |  |  | **Within full sample** | | **Within those not using other products^2^** | |
|  | **%** | **n** | **%** | **n** | **%** | **N** |
| **Age group (years)** |  |  |  |  |  |  |
| 16 - 17 | 47.7 | 18701 | 26.0 | 4853 | 23.3 | 3765 |
| 18 - 19 | 52.3 | 20513 | 32.8 | 6735 | 28.4 | 4571 |
| **Sex** |  |  |  |  |  |  |
| Male | 32.5 | 12758 | 26.4 | 3366 | 21.5 | 2223 |
| Female | 67.5 | 26456 | 31.1 | 8222 | 27.8 | 6113 |
| **Country** |  |  |  |  |  |  |
| Canada | 32.1 | 12592 | 26.4 | 3318 | 22.4 | 2306 |
| England | 29.9 | 11742 | 30.3 | 3557 | 26.9 | 2695 |
| US | 37.9 | 14880 | 31.7 | 4713 | 27.9 | 3335 |
| **Race/Ethnicity** |  |  |  |  |  |  |
| White | 57.4 | 22521 | 28.0 | 6308 | 24.1 | 4433 |
| Everyone else | 42.6 | 16693 | 31.6 | 5280 | 28.1 | 3903 |
| **Perceived family socio-economic situation** |  |  |  |  |  |  |
| Not meeting basic expenses | 3.2 | 1248 | 40.9 | 510 | 34.8 | 280 |
| Just meeting basic expenses | 22.6 | 8880 | 35.0 | 3109 | 29.8 | 2020 |
| Meeting needs with a little left over | 33.9 | 13312 | 28.9 | 3841 | 25.5 | 2852 |
| Living comfortably | 34.6 | 13576 | 26.6 | 3615 | 24.0 | 2760 |
| Don’t know/refused | 5.6 | 2198 | 23.3 | 513 | 21.3 | 424 |
| **Past 30-day smoking and/or vaping** |  |  |  |  |  |  |
| Vaped only | 8.8 | 3460 | 40.7 | 1409 | 34.1 | 619 |
| Smoked only | 4.6 | 1808 | 41.7 | 754 | 32.7 | 302 |
| Smoked and vaped | 5.4 | 2129 | 50.7 | 1080 | 35.9 | 263 |
| Neither | 81.1 | 31817 | 26.2 | 8345 | 24.8 | 7152 |
| **Lifetime/current smoking** |  |  |  |  |  |  |
| ≥20 days in past 30 days | 2.7 | 1046 | 54.2 | 567 | 41.0 | 157 |
| <20 days in past 30 days | 1.6 | 647 | 46.7 | 302 | 35.8 | 92 |
| Smoked in the past | 1.1 | 417 | 42.0 | 175 | 34.3 | 74 |
| Experimented in the past | 26.1 | 10245 | 35.2 | 3602 | 29.0 | 1971 |
| Never smoked | 68.5 | 26859 | 25.8 | 6942 | 24.5 | 6042 |
| **Lifetime/current vaping** |  |  |  |  |  |  |
| ≥20 days in past 30 days | 4.7 | 1857 | 49.8 | 924 | 40.9 | 296 |
| <20 days in past 30 days | 6.3 | 2478 | 43.7 | 1083 | 33.6 | 378 |
| Vaped in the past | 6.1 | 2389 | 37.3 | 890 | 32.0 | 462 |
| Experimented in the past | 21.6 | 8480 | 32.4 | 2750 | 29.7 | 1936 |
| Never vaped | 61.2 | 24010 | 24.7 | 5941 | 23.4 | 5264 |
| **Other inhaled product use in past 30 days ^1^** |  |  |  |  |  |  |
| None | 82.3 | 32274 | 25.8 | 8336 | n/a | n/a |
| 1 type of product | 14.1 | 5538 | 43.6 | 2416 | n/a | n/a |
| 2 or 3 types of products | 3.6 | 1402 | 59.6 | 836 | n/a | n/a |
| **Survey wave** |  |  |  |  |  |  |
| August 2020 | 34.3 | 13445 | 29.9 | 4020 | 26.3 | 2928 |
| February/March 2021 | 33.2 | 13010 | 28.6 | 3719 | 24.8 | 2653 |
| August/September 2021 | 32.5 | 12759 | 30.2 | 3849 | 26.4 | 2755 |

Note: Columns 2 and 3 (Sample description) show the proportion and number reporting each characteristic such as being aged 16 or 17 and the subgroups will add up to 100% (allowing for rounding). Columns 4 to 7 (Reported any respiratory symptoms) show the proportion and number within each subgroup who reported symptoms, e.g. the proportion of those aged 16 or 17 who reported experiencing any respiratory symptoms in the past week; therefore, the subgroups will not add up to 100% in these columns.

^1^ Proportion reporting past-30-day use in the full sample.

^2^ N=32,274. This is the sample of people who reported no use of heated tobacco, smoking or vaping cannabis, or other combustible tobacco products.

**Table S2. Respiratory symptoms in the past week broken down by other product use, weighted n (%).**

|  | **No other product used** | **1 other product used** | **2 or 3 other products used** |
| --- | --- | --- | --- |
| Any symptom | 24.4 (8049) | 42.7 (2202) | 58.5 (730) |
| Shortness of breath | 8.2 (2705) | 17.4 (898) | 27.6 (344) |
| Wheezing | 1.8 (582) | 5.7 (296) | 11.2 (140) |
| Chest pain | 8.7 (2880) | 15.6 (806) | 21.2 (264) |
| Phlegm | 7.1 (2331) | 14.1 (727) | 22.9 (285) |
| Cough | 13.5 (4461) | 27.1 (1398) | 38.8 (484) |

**Table S3.** **Vaping product characteristics and respiratory symptoms by characteristic among those who had vaped in the past 30 days (unweighted data).**

| **Vaping product characteristic** | **Sample description** | | **Reported any respiratory symptoms** | | | |
| --- | --- | --- | --- | --- | --- | --- |
|  |  |  | **Within full sample** | | **Within those not using other products** | |
|  | **%** | **n** | **%** | **n** | **%** | **n** |
| **Nicotine type ^1^** |  |  |  |  |  |  |
| Non-salt, unknown | 79.3 | 3681 | 42.2 | 1553 | 32.7 | 590 |
| Salt | 20.7 | 963 | 55.7 | 536 | 48.8 | 141 |
|  |  |  |  |  |  |  |
| **Flavour type** |  |  |  |  |  |  |
| Fruit | 50.4 | 2340 | 41.9 | 980 | 33.9 | 399 |
| Multiple | 24.9 | 1157 | 53.2 | 616 | 44.1 | 165 |
| Menthol or mint | 12.5 | 581 | 42.0 | 244 | 29.3 | 80 |
| Tobacco (incl. menthol tobacco) | 4.9 | 226 | 38.1 | 86 | 24.8 | 25 |
| Other, unknown | 4.8 | 223 | 49.8 | 111 | 37.8 | 42 |
| Sweets | 2.5 | 117 | 44.4 | 52 | 34.5 | 20 |
|  |  |  |  |  |  |  |
| **Device type** |  |  |  |  |  |  |
| Pre-filled cartridge/pod | 36.9 | 1715 | 43.4 | 744 | 33.8 | 270 |
| Tank | 29.0 | 1347 | 43.8 | 590 | 37.2 | 265 |
| Disposable | 18.2 | 847 | 46.0 | 390 | 30.9 | 104 |
| Multiple, unknown | 15.8 | 735 | 49.7 | 365 | 37.9 | 92 |

Note: Columns 2 and 3 (Sample description) show the proportion and number reporting each characteristic such as using nicotine salts and the subgroups will add up to 100% (allowing for rounding). Columns 4 to 7 (Reported any respiratory symptoms) show the proportion and number within each subgroup who reported symptoms, e.g. the proportion of those using nicotine salts who reported experiencing any respiratory symptoms in the past week; therefore, the subgroups will not add up to 100% in these columns.

**^1^** Unweighted n=4,644 for full sample, n=2,093 for those not using other products (those who reported no use of heated tobacco, smoking or vaping cannabis, or other combustible tobacco products).

## Sensitivity analyses and supplementary analyses

1. Sensitivity analyses - Respondents who had not used other inhaled products (heated tobacco, cannabis, other combustible tobacco products)
2. Sensitivity analyses - Including whether respondents had Covid in past 2 weeks, 2021 waves only
3. Supplementary analyses – Individual symptoms

### Hypothesis 1. Current vaping will be associated with lower odds of respiratory symptoms than current smoking and higher odds than not smoking or vaping.

### Hypothesis 2. More frequent vaping will be associated with higher odds of respiratory symptoms than less frequent vaping, independent of smoking status.

**Table S4. Associations between past-30-day smoking and/or vaping, lifetime/current vaping, number of days vaped in the past 30 days and any respiratory symptoms (weighted data).**

|  | **1. No other products used** | | **2. Covid yes/no included** | |
| --- | --- | --- | --- | --- |
|  | **Adjusted OR (95 % CI)** | **p-value** | **Adjusted OR (95 % CI)** | **p-value** |
| **Past 30-day smoking and/or vaping ^1^** |  |  |  |  |
| Vaped only | Ref |  | Ref |  |
| Smoked only | 0.93 (0.78 - 1.12) | 0.448 | 0.97 (0.83 - 1.15) | 0.781 |
| Smoked and vaped | 1.06 (0.87 - 1.29) | 0.541 | 1.20 (1.03 – 1.39) | 0.019 |
| Neither | 0.63 (0.56 - 0.69) | <0.001 | 0.67 (0.61 – 0.74) | <0.001 |
|  |  |  |  |  |
| **Lifetime/current vaping ^2^** |  |  |  |  |
| ≥20 days in past 30 days | 2.31 (1.95 – 2.73) | <0.001 | 2.06 (1.79 – 2.37) | <0.001 |
| <20 days in past 30 days | 1.57 (1.36 – 1.81) | <0.001 | 1.49 (1.31 – 1.69) | <0.001 |
| Vaped in the past | 1.35 (1.19 – 1.54) | <0.001 | 1.21 (1.07 – 1.38) | 0.003 |
| Experimented in the past | 1.43 (1.33 – 1.53) | <0.001 | 1.21 (1.12 – 1.31) | <0.001 |
| Never vaped | Ref |  | Ref |  |
|  |  |  |  |  |
| **Number days vaped in past 30 days ^3^** |  |  |  |  |
| Per additional day | 1.02 (1.01 – 1.03) | <0.001 | 1.02 (1.01 – 1.03) | <0.001 |

**^1^** Adjusted analysis included age group, sex, country, ethnicity, perceived family socio-economic status, wave, and for analysis 2 also use of other inhaled products. Unweighted n=32,274 in analysis 1 and 25,769 in analysis 2.

**^2^** Adjusted analysis included age group, sex, country, ethnicity, perceived family socio-economic status, lifetime/current smoking, wave, and for analysis 2 also use of other inhaled products. Unweighted n=32,274 in analysis 1 and 25,769 in analysis 2.

**^3^** Adjusted analysis included age group, sex, country, ethnicity, perceived family socio-economic status, lifetime/current smoking, wave, and for analysis 2 also use of other inhaled products. Unweighted n=2,093 in analysis 1 and 3,243 in analysis 2.

**Table S5. Associations between past-30-day smoking and vaping, lifetime/current vaping, number of days vaped in the past 30 days and individual respiratory symptoms (weighted data).**

|  | **Adjusted OR (95% CI), p** | | | | |
| --- | --- | --- | --- | --- | --- |
| **Past 30-day smoking and/or vaping ^1^** | **Shortness of breath** | **Wheezing** | **Chest pain** | **Phlegm** | **Cough** |
| Vaped only | Ref | Ref | Ref | Ref | Ref |
| Neither | 0.66 (0.59 - 0.74), <0.001 | 0.75 (0.61 - 0.92), 0.006 | 0.88 (0.79 - 0.99), 0.033 | 0.72 (0.64 - 0.81), <0.001 | 0.59 (0.54 - 0.64), <0.001 |
| Smoked only | 0.96 (0.81 - 1.13), 0.599 | 1.18 (0.89 - 1.58), 0.250 | 0.78 (0.64 - 0.94), 0.011 | 1.09 (0.91 - 1.31), 0.338 | 1.00 (0.87 - 1.15), 0.980 |
| Smoked and vaped | 1.25 (1.08 - 1.46), 0.004 | 1.67 (1.30 - 2.14), <0.001 | 1.24 (1.05 - 1.46), 0.010 | 1.31 (1.12 - 1.54), 0.001 | 1.44 (1.26 - 1.63), <0.001 |
|  |  |  |  |  |  |
| **Lifetime/current vaping ^2^** |  |  |  |  |  |
| ≥20 days in past 30 days | 1.89 (1.62 – 2.20), <0.001 | 1.19 (0.90 – 1.57), 0.212 | 1.64 (1.40 – 1.92), <0.001 | 1.85 (1.58 – 2.17), <0.001 | 2.29 (2.02 – 2.59), <0.001 |
| <20 days in past 30 days | 1.67 (1.46 – 1.92), <0.001 | 1.31 (1.03 – 1.67), 0.029 | 1.47 (1.27 – 1.70), <0.001 | 1.53 (1.31 – 1.77), <0.001 | 1.65 (1.47 – 1.85), <0.001 |
| Vaped in the past | 1.30 (1.12 – 1.50), <0.001 | 1.12 (0.86 – 1.45), 0.394 | 1.44 (1.24 – 1.66), <0.001 | 1.59 (1.38- 1.84), <0.001 | 1.30 (1.15 – 1.460, <0.001 |
| Experimented in the past | 1.24 (1.13 – 1.36), <0.001 | 1.14 (0.96 – 1.36), 0.147 | 1.29 (1.18 – 1.41), <0.001 | 1.27 (1.15 – 1.40), <0.001 | 1.33 (1.23 – 1.43), <0.001 |
| Never vaped | Ref | Ref | Ref | Ref | Ref |
|  |  |  |  |  |  |
| **Number days vaped ^2^** |  |  |  |  |  |
| Per additional day | 1.01 (1.01 - 1.02), <0.001 | 1.00 (0.99 - 1.01), 0.707 | 1.01 (1.00 - 1.02), 0.013 | 1.01 (1.00 - 1.02), 0.039 | 1.02 (1.01 - 1.02), <0.001 |

^1^ Adjusted analysis included age group, sex, country, ethnicity, perceived family socio-economic status, use of other inhaled products, wave. Unweighted n= 39,214.

^2^ Adjusted analysis included age group, sex, country, ethnicity, perceived family socio-economic status, use of other inhaled products, wave, lifetime/current smoking. Unweighted n= 39,214.

^3^ Adjusted analysis included age group, sex, country, ethnicity, perceived family socio-economic status, use of other inhaled products, wave, lifetime/current smoking. Unweighted n=4,644.

### Hypothesis 3. Vaping nicotine salts will be associated with higher odds of respiratory symptoms compared with vaping other forms of nicotine; this will at least partly be explained by frequency of vaping.

### Hypothesis 4. Different types of flavours or types of devices will not be associated with respiratory symptoms independently of frequency, nicotine type and smoking status.

**Table S6. Associations between vaping characteristics and any respiratory symptoms (weighted data).**

|  | **1. No other products used** | | **2. Covid yes/no included** | |
| --- | --- | --- | --- | --- |
| **Characteristic, past 30 days** | **Adjusted OR (95 % CI)** | **p-value** | **Adjusted OR (95 % CI)** | **p-value** |
| **Nicotine type^1^** |  |  |  |  |
| Non-salt, unknown | Ref |  | Ref |  |
| Salt | 1.92 (1.46 – 2.52) | <0.001 | 1.45 (1.20 – 1.76) | <0.001 |
|  |  |  |  |  |
| **Flavour type ^2^** |  |  |  |  |
| Tobacco | Ref |  | Ref |  |
| Menthol or mint | 0.89 (0.54 - 1.47) | 0.651 | 1.43 (0.94 – 2.16) | 0.095 |
| Fruit | 1.19 (0.76 - 1.87) | 0.445 | 1.58 (1.08 – 2.30) | 0.017 |
| Sweets | 1.29 (0.64 - 2.57) | 0.474 | 1.84 (1.06 – 3.19) | 0.030 |
| Other, unknown | 1.35 (0.75 – 2.44) | 0.322 | 2.17 (1.31 – 3.60) | 0.003 |
| Multiple | 1.71 (1.06 - 2.76) | 0.029 | 1.80 (1.22 - 2.65) | 0.003 |
|  |  |  |  |  |
| **Device type ^2^** |  |  |  |  |
| Pre-filled cartridge/pod | Ref |  | Ref |  |
| Disposable | 1.05 (0.78 - 1.41) | 0.760 | 1.05 (0.84 - 1.32) | 0.649 |
| Tank | 1.06 (0.84 - 1.34) | 0.634 | 0.91 (0.75 - 1.12) | 0.378 |
| Multiple, unknown | 1.22 (0.89 - 1.67) | 0.216 | 0.98 (0.78 – 1.24) | 0.886 |

Unweighted n=2,093 in analysis 1 and n=3,243 in analysis 2.

**^1^** Adjusted analysis included age group, sex, country, ethnicity, perceived family socio-economic status, lifetime/current smoking, number of days vaped, wave, and for analysis 2 also use of other inhaled products.

^2^ Adjusted analysis included age group, sex, country, ethnicity, perceived family socio-economic status, lifetime/current smoking, number of days vaped, nicotine type, wave, and for analysis 2 also use of other inhaled products.

**Table S7. Associations between vaping characteristics and individual respiratory symptoms (weighted data).**

|  | **Adjusted OR (95% CI), p** |  |  |  |  |
| --- | --- | --- | --- | --- | --- |
|  | **Shortness of breath** | **Wheezing** | **Chest pain** | **Phlegm** | **Cough** |
| **Nicotine type^1^** |  |  |  |  |  |
| Non-salt, unknown | Ref | Ref | Ref | Ref | Ref |
| Salt | 1.52 (1.26 - 1.84), <0.001 | 1.50 (1.10 - 2.04), 0.010 | 1.37 (1.11 - 1.69), 0.003 | 1.71 (1.41 - 2.08), <0.001 | 1.25 (1.06 - 1.47), 0.008 |
|  |  |  |  |  |  |
| **Flavour type ^2^** |  |  |  |  |  |
| Tobacco | Ref | Ref | Ref | Ref | Ref |
| Menthol or mint | 0.70 (0.46 - 1.06), 0.093 | 0.84 (0.42 - 1.67), 0.621 | 1.02 (0.62 - 1.67), 0.941 | 0.95 (0.62 - 1.46), 0.817 | 1.74 (1.20 - 2.52), 0.003 |
| Fruit | 0.82 (0.57 -1.18), 0.283 | 0.88 (0.49 - 1.57), 0.660 | 1.38 (0.89 - 2.14), 0.145 | 0.98 (0.67 - 1.44), 0.920 | 1.76 (1.25 - 2.46), 0.001 |
| Sweets | 0.99 (0.55 - 1.77), 0.966 | 0.43 (0.12 - 1.57), 0.201 | 1.09 (0.54 - 2.19), 0.812 | 1.18 (0.64 - 2.17), 0.589 | 1.70 (1.01 - 2.87), 0.046 |
| Other, unknown | 1.14 (0.71 – 1.85), 0.586 | 1.69 (0.82 - 3.49), 0.157 | 1.94 (1.13 - 3.34), 0.017 | 0.79 (0.46 - 1.39), 0.424 | 2.42 (1.57 – 3.73), <0.001 |
| Multiple | 1.06 (0.73 -1.53), 0.768 | 1.27 (0.71 - 2.30), 0.422 | 1.35 (0.86 - 2.12), 0.188 | 1.18 (0.80 - 1.75), 0.403 | 2.15 (1.52 - 3.04), <0.001 |
|  |  |  |  |  |  |
| **Device type ^2^** |  |  |  |  |  |
| Pre-filled cartridge/pod | Ref | Ref | Ref | Ref | Ref |
| Disposable | 1.31 (1.04 - 1.64), 0.024 | 1.42 (0.97 - 2.08), 0.075 | 1.39 (1.08 - 1.78), 0.011 | 1.37 (1.07 - 1.75), 0.012 | 1.05 (0.87 - 1.29), 0.599 |
| Tank | 0.92 (0.74 - 1.14), 0.444 | 0.80 (0.55 - 1.16), 0.240 | 1.19 (0.95 - 1.51), 0.14 | 1.22 (0.98 - 1.52), 0.081 | 0.95 (0.79 - 1.13), 0.526 |
| Multiple, unknown | 1.14 (0.90 - 1.45), 0.564 | 1.31 (0.89 – 1.91), 0.173 | 1.50 (1.16 - 1.93), 0.002 | 1.08 (0.84 - 1.40), 0.553 | 0.97 (0.79 - 1.19), 0.778 |

^1^ Adjusted analysis included age group, sex, country, ethnicity, perceived family socio-economic status, use of other inhaled products, wave, lifetime/current smoking, number of days vaped. Unweighted n = 4,644.

^2^ Adjusted analysis included age group, sex, country, ethnicity, perceived family socio-economic status, use of other inhaled products, wave, lifetime/current smoking, number of days vaped, nicotine type. Unweighted n= 4,644.

## Sensitivity, interaction and supplementary analyses for Hypothesis 5 - There will be no country differences and no interactions between country and vaping, or country and vaping characteristics on respiratory symptoms.

**Table S8. Sensitivity analysis. Associations between country and any respiratory symptoms for the full sample and those who had vaped in the past 30 days (weighted data).**

|  | **1. No other products used** | | **2. Covid yes/no included** | |
| --- | --- | --- | --- | --- |
|  | **Adjusted OR (95 % CI)** | **p-value** | **Adjusted OR (95 % CI)** | **p-value** |
| **Country, full sample ^1^** |  |  |  |  |
| England | Ref |  | Ref |  |
| Canada | 0.78 (0.73 – 0.84) | <0.001 | 0.86 (0.80 – 0.92) | <0.001 |
| US | 1.09 (1.03 – 1.16) | 0.004 | 1.17 (1.09 – 1.25) | <0.001 |
|  |  |  |  |  |
| **Country, vaped in past 30 days ^2^** |  |  |  |  |
| England | Ref |  | Ref |  |
| Canada | 0.99 (0.78 – 1.26) | 0.947 | 1.06 (0.88 – 1.28) | 0.522 |
| US | 0.96 (0.77 - 1.20) | 0.715 | 0.99 (0.82 – 1.20) | 0.943 |

**^1^** Unweighted n=32,274 in analysis 1 and n=25,769 in analysis ^2^ Unweighted n=2,093 in analysis 1 and n=3,243 in analysis 2. Adjusted analysis included age group, sex, ethnicity, perceived family socio-economic status, wave, and for analysis 2 also use of other inhaled products.

**Table S9. Interaction models for country; primary outcome; models were separate from each other; reference country is England (weighted data).**

|  | **Adjusted OR (95 % CI)** | **p-value** |
| --- | --- | --- |
| **Country * Past 30-day smoking and/or vaping** (Reference vaped only) |  | <0.001 |
| Canada * Smoked only | 0.75 (0.56 – 1.04) | 0.084 |
| Canada * Smoked and vaped | 0.97 (0.72 – 1.29) | 0.817 |
| Canada * Neither | 0.71 (0.60 – 0.87) | 0.001 |
| US * Smoked only | 0.99 (0.70 – 1.41) | 0.970 |
| US * Smoked and vaped | 1.07 (0.79 – 1.44) | 0.686 |
| US * Neither | 1.01 (0.83 – 1.23) | 0.897 |
|  |  |  |
| **Country * Lifetime/current vaping** (Reference never vaped) |  | <0.001 |
| Canada * ≥20 days in past 30 days | 1.60 (1.22 – 2.10) | <0.001 |
| Canada * <20 days in past 30 days | 0.97 (0.77 – 1.21) | 0.767 |
| Canada * Vaped in the past | 0.88 (0.70 – 1.11) | 0.270 |
| Canada * Experimented in the past | 0.87 (0.75 – 1.00) | 0.048 |
| US * ≥20 days in past 30 days | 0.98 (0.75 – 1.27) | 0.854 |
| US * <20 days in past 30 days | 0.70 (0.57 – 0.87) | 0.001 |
| US * Vaped in the past | 0.63 (0.50 – 0.78) | <0.001 |
| US * Experimented in the past | 0.82 (0.72 – 0.93) | 0.003 |
|  |  |  |
| **Country * Nicotine type** (Reference non-salt, unknown) |  | 0.003 |
| Canada * Yes | 1.38 (0.94 – 2.02) | 0.102 |
| US * Yes | 0.77 (0.52 – 1.12) | 0.173 |
|  |  |  |
| **Country * Flavour type** (Reference tobacco/tobacco menthol) |  | 0.257 |
| Canada * Menthol or mint | 1.16 (0.52 – 2.58) | 0.718 |
| Canada * Fruit | 0.73 (0.37 – 1.44) | 0.359 |
| Canada * Sweets | 0.50 (0.17 – 1.47) | 0.205 |
| Canada * Other, unknown | 0.37 (0.15 – 0.96) | 0.040 |
| Canada * Multiple | 0.93 (0.45 – 1.92) | 0.848 |
| US * Menthol or mint | 1.13 (0.53 – 2.43) | 0.751 |
| US * Fruit | 0.92 (0.48 – 1.80) | 0.816 |
| US * Sweets | 1.24 (0.42 – 3.69) | 0.702 |
| US * Other, unknown | 0.54 (0.21 – 1.38) | 0.197 |
| US * Multiple | 1.14 (0.56 – 2.29) | 0.720 |
|  |  |  |
| **Country * Device type** (Reference pre-filled cartridge/pod) |  | 0.226 |
| Canada * Disposable | 0.63 (0.38 – 1.06) | 0.083 |
| Canada * Tank | 0.97 (0.66 – 1.42) | 0.868 |
| Canada * Multiple, unknown | 1.25 (0.76 – 2.05) | 0.389 |
| US * Disposable | 0.65 (0.41 – 1.02) | 0.059 |
| US * Tank | 0.86 (0.57 – 1.28) | 0.451 |
| US * Multiple, unknown | 1.30 (0.80 – 2.14) | 0.293 |

Unweighted n=39,214 for past 30-day smoking and/or vaping and lifetime/current vaping; unweighted n=4,644 for nicotine type, flavour type and device type. Adjusted analysis included age group, sex, country, ethnicity, perceived family socio-economic status, wave.

**Table S10. Supplementary analysis. Associations between country and individual respiratory symptoms for the full sample and those who had vaped in the past 30 days (weighted data).**

|  | **Adjusted OR (95% CI), p** | | | | |
| --- | --- | --- | --- | --- | --- |
|  | **Shortness of breath** | **Wheezing** | **Chest pain** | **Phlegm** | **Cough** |
| **Country, full sample ^1^** |  |  |  |  |  |
| England | Ref | Ref | Ref | Ref | Ref |
| Canada | 0.87 (0.80 – 0.95), 0.002 | 0.79 (0.68 – 0.93), 0.004 | 0.81 (0.75 – 0.89), <0.001 | 0.83 (0.76 – 0.92), <0.001 | 0.92 (0.85 – 0.98), 0.013 |
| US | 1.01 (0.93 – 1.09), 0.845 | 0.82 (0.71 – 0.96), 0.011 | 1.12 (1.04 – 1.21), 0.005 | 1.04 (0.96 – 1.14), 0.332 | 1.11 (1.04 – 1.18), 0.002 |
|  |  |  |  |  |  |
| **Country, vaped in past 30 days ^2^** |  |  |  |  |  |
| England | Ref | Ref | Ref | Ref | Ref |
| Canada | 1.08 (0.88 – 1.32), 0.466 | 0.84 (0.61 – 1.14), 0.253 | 0.94 (0.76 – 1.18), 0.608 | 1.00 (0.82 – 1.23), 0.973 | 1.07 (0.91 – 1.27), 0.417 |
| US | 0.94 (0.77 – 1.15), 0.535 | 0.48 (0.34 – 0.67), <0.001 | 0.99 (0.80 – 1.23), 0.953 | 0.85 (0.69 – 1.04), 0.111 | 0.97 (0.83 – 1.14), 0.725 |

^1^ n=39,214. Adjusted analysis included age group, sex, ethnicity, perceived family socio-economic status, wave.

^2^ n=4,644. Adjusted analysis included age group, sex, ethnicity, perceived family socio-economic status, wave.
